# Building capacity to treat childhood cancer in Papua New Guinea: ‘It’s a multidisciplinary village’

**DOI:** 10.7189/jogh.15.03008

**Published:** 2025-02-14

**Authors:** Trisha A Soosay Raj, Jayne Harrison, Claire E Wakefield, Benjamin Daur, Ben Felmingham, Michele Casey, Jordana K McLoone, Michael Sullivan, Sandra E Staffieri, Gwenda Anga

**Affiliations:** 1 Oncology Services Group, Queensland Children’s Hospital, South Brisbane Queensland, Australia; 2 Children’s Health Queensland Clinical Unit, University of Queensland, Brisbane, Queensland, Australia; 3 Children’s Cancer Centre, The Royal Children’s Hospital, Melbourne, Victoria, Australia; 4 Murdoch Children’s Research Institute, Global Health, Melbourne, Victoria, Australia; 5 School of Clinical Medicine, Faculty of Medicine, UNSW Sydney, Randwick, New South Wales, Australia; 6 Behavioural Sciences Unit, Kids Cancer Centre, Sydney Children’s Hospital, Randwick, New South Wales, Australia; 7 Port Moresby General Hospital, Port Moresby, Papua New Guinea; 8 Cardiac Regeneration Laboratory, Murdoch Children’s Research Institute, Melbourne, Victoria, Australia; 9 Cancer Centre for Children, The Children’s Hospital at Westmead, Sydney, New South Wales, Australia; 10 Murdoch Children’s Research Institute, Department of Paediatrics, University of Melbourne, Melbourne, Victoria, Australia; 11 Centre for Eye Research Australia, Royal Victorian Eye and Ear Hospital, and University of Melbourne, Melbourne, Victoria, Australia; 12 Department of Ophthalmology, Royal Children’s Hospital, Melbourne, Victoria, Australia

## Abstract

Childhood cancer outcomes in low- and middle-income countries are impacted by the presentation of advanced disease and limited diagnostic and treatment resources. Papua New Guinea is highly populated with significant health coverage and workforce difficulties, in addition to unique geographical and political challenges affecting childhood cancer care. With improvements in communicable disease management, childhood cancer care has become an emerging need, managed by a dedicated service in Port Moresby General Hospital (PMGH). A longstanding partnership between PMGH and the International Society of Paediatric Oncology Oceania has facilitated the development of a cancer registry, education/training, research, and technical support. We describe an in-country visit comprising a tailored childhood cancer workshop for health care workers, with advocacy and collaboration efforts. Goals included education, childhood cancer registry implementation, clinical support, stakeholder engagement and supply of practical resources. Outcomes include enhanced nursing capacity with the establishment of a national oncology nurses association for peer support and ongoing educational opportunities. Key learnings include identifying palliative care as an unmet need, unique cultural aspects allowing for future targeted education, further collaboration on adapted treatment regimens, and formalised multidisciplinary meetings for enhanced practice. This partnership demonstrates the positive effect of strong local champions partnering with supportive peer relationships in global oncology.

Improving the outcomes for children with cancer in low- and middle-income countries (LMICs) presents a great challenge for health care professionals and their health services. Many factors contribute to the poorer outcomes for childhood cancer in LMICs, including advanced disease at diagnosis, delays in referral and access to cancer care, and limited resources for the timely diagnosis and treatment of cancer [1]. These challenges are particularly acute in the Pacific Islands and across Oceania, where multiple small-island developing states and territories face additional difficulties due to their geographic isolation, relatively small populations, and small generalist health care workforces, who have limited opportunities for specialist professional education and training. Compounding the capacity limitations of their health services, countries in the Pacific Islands and Oceania have many natural climate events that appear to be becoming more frequent and have small, fragile economies with high remittance migration and family separation [2]. The World Health Organization (WHO) has recognised these challenges with a special initiative focusing on small-island developing states as a global health priority [2]. With a population of over 10 million, Papua New Guinea is the largest and most populous Pacific Island country. Yet, it has the lowest level of universal health coverage in Oceania (the third lowest worldwide) [3] and very low health workforce staffing with 0.06 physicians and 0.5 nurses per 1000 people [4]. Over 80% of the population lives rurally, with limited access by road and no rail network; many regions are only accessible by sea or air. However, over the last 30 years, improving child health in Papua New Guinea has been a national health priority, with universal access to childhood immunisations, improved public health and access to care for childhood infections. Globally, improved infant health has seen childhood cancer emerge as an important unmet health care need, and this is now evident in Papua New Guinea [2].

No national childhood cancer incidence data are yet available for Papua New Guinea. However, an estimate of annual childhood cancer incidence, based on a total childhood population of approximately four million, at 120 cases per million children, suggests that >400 children per year will develop cancer in Papua New Guinea. Global estimates suggest at least 50% of children with cancer are undiagnosed, and in Papua New Guinea, we estimate more than 70% of children with cancer are undiagnosed [5]. To address this emerging child cancer burden, the Papua New Guinea Ministry of Health cancer control policy has identified a need for better integration of paediatric cancer care into the national cancer control plan, specifically in early detection, referral, and capacity building [6].

## PAPUA NEW GUINEA CHILDREN’S CANCER SERVICE

To address the emerging need for child cancer care in Papua New Guinea, a dedicated child cancer service was established in 2015 based in the national hospital, Port Moresby General Hospital, in the capital city of Port Moresby [6]. The 12-bed childhood cancer ward is staffed by two lead Oncology Paediatricians (one completed a 12-month paediatric oncology observership in Melbourne, Australia, and one completed in-country training), six dedicated paediatric nurses, and several chaplains providing psychosocial support. The service now has access to some but not all of the WHO Essential Cancer Chemotherapy medicines and, in collaboration with the International Society of Paediatric Oncology (SIOP) Oceania colleagues, has developed and implemented Papua New Guinea-specific adapted treatment regimens for the six WHO Global Initiative for Childhood Cancer index cancers.

An initial audit following the establishment of the Port Moresby General Hospital (PMGH) child cancer service for the two-year period (January 2016–January 2018) identified 61 children (aged <15 years) referred for diagnosis and treatment. Of these, 15 died on therapy (24%) and 14 (23%) successfully completed treatment. A further 21 elected not to commence cancer treatment (34%), four (6%) abandoned treatment and left the hospital, and seven (11%) elected out-of-county treatment [7]. A second, more contemporary audit of diagnosis and treatment outcome is pending an analysis of data from the recently established Papua New Guinea Children’s Cancer Registry. While these initial data represent a significant outcome gap relative to neighbouring high-income countries (Australia and New Zealand), the PMGH team has achieved a significant improvement in access to cancer care and outcomes over time [8]. This initial audit demonstrated the need for dedicated child cancer care in Papua New Guinea and has established a service base for the future improvement in cancer outcomes in keeping with the WHO Global Initiative for Childhood Cancer CureAll strategy. Moreover, having established the service, the Papua New Guinea health care team has successfully advocated for improved access and availability to treatment for a broader range of child cancer diagnoses, enhanced supportive care, and improved multidisciplinary staff training. The PMGH team has also identified the need to improve family support and education.

Despite these strengths, the team faces significant challenges. With the increased awareness of childhood cancer in Papua New Guinea, the number of new case referrals has risen significantly (especially post-COVID), and with this has come the demand for the service and the health care team.

Additionally, access to resources for the accurate and timely diagnosis and treatment of children with cancer in Papua New Guinea remains limited. Currently, there is no cancer-specific pathology service available. Access to diagnostic biopsy for solid tumours is very limited (no interventional radiology), and there are long delays (up to six months) for basic histopathology. There is currently no access to immunohistochemistry, flow cytometry, or molecular diagnosis [6]. There is also limited capability and no dedicated facility to perform common diagnostic procedures such as lumbar punctures with intrathecal chemotherapy, bone marrow aspirates and trephine biopsies. PMGH does not have a dedicated paediatric intensive care unit, but there is access to a mixed high-dependency unit. There is currently no paediatric palliative care program.

The Papua New Guinea-adapted treatment protocols are adapted to a SIOP-Paediatric Oncology for Developing Countries (Global Health Network) resource level two setting for treatment with curative intent without access to radiotherapy. Papua New Guinea is developing a new national radiotherapy facility, so radiotherapy is currently unavailable. Access to chemotherapy also remains a challenge. 14 of the 65 antineoplastic and immunomodulators listed in the WHO Model List of Essential Medicines are available at PMGH [6], with some medicines available for private purchase. There is no dedicated chemotherapy preparation facility, so chemotherapy preparation and dispensing are currently delegated to ward nursing and junior medical staff; this is an area to be addressed to ensure Papua New Guinea can meet international standards for chemotherapy preparation and handling [6]. Most families referred to our PMGH cancer service have very limited financial resources, especially those from remote/rural regions. The service also lacks suitable long-term accommodation for parents who stay with their children for long periods in the 12-bed ward. These family financial and accommodation constraints lead to disrupted care and treatment non-completion [6].

## BUILDING CAPACITY IN PAPUA NEW GUINEA THROUGH SUSTAINED PARTNERSHIPS

Building capacity for subspecialist medical and nursing care within a generalist workforce is a major challenge for sustained improvement in child cancer services in countries such as Papua New Guinea. Fortunately, the Papua New Guinea paediatric oncology program can draw upon external support and expertise for training and clinical service development through partnerships in Australia. Since 2014, the oncology teams at the Royal Children’s Hospital (Melbourne) and Queensland Children’s Hospital (Brisbane) Australia have provided sustained support for case clinical conference discussions, continuing education, and nursing training. The Papua New Guinea program is also supported by SIOP Oceania, one of six continental branches of SIOP. SIOP Oceania advocates for children and adolescents with cancer across the Oceania region, including developing and sharing technical expertise to improve childhood cancer services, promoting research to improve outcomes, and developing education and training opportunities. SIOP Oceania members and collaborators have recently developed and supported the implementation of a national Papua New Guinea child cancer registry developed in the Research Electronic Data Capture tool (REDcap), version 13.3.0 (Vanderbilt University, Nashville, TN, USA) database and hosted at PMGH. More recently, the SIOP Oceania nursing group has supported the formation of a Papua New Guinea Oncology Nursing Group who communicate over a national WhatsApp group and hold monthly Zoom meetings for nursing education and training. This group, supported by SIOP Oceania nursing, has identified gaps in care and resources, such as access to personal protective equipment education and has provided basic equipment.

At the invitation of the national lead oncology paediatrician, a delegation from SIOP Oceania visited Papua New Guinea in February 2023. The visit goals were to assess the current in-country resources for child cancer care, to co-develop and deliver an interprofessional, interactive, in-country childhood cancer training workshop, and to support child cancer advocacy by meeting with senior members of the Papua New Guinea Ministry of Health and the in-country WHO team. The SIOP Oceania delegation included eight volunteers (two paediatric oncologists, two paediatric oncology nurses, an oncology pharmacist, a medical psychologist, a retinoblastoma care co-ordinator, and a program manager).

Prior to the visit, the delegation reviewed available professional education resources for content, language, and appropriateness and tailored the workshop delivery with the local team. Although several high-quality paediatric oncology education programs developed for LMICs were available, their content was generally too detailed and advanced for non-specialist nursing and largely not applicable to the current Papua New Guinea context. For instance, available programs assumed patients had access to central lines for chemotherapy and more advanced access to cancer chemotherapy and supportive care.

**Figure 1:**
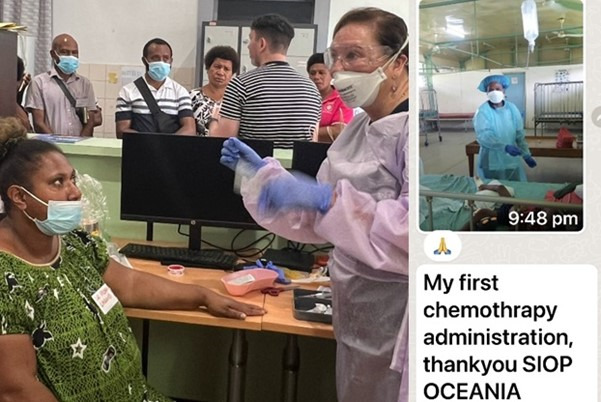
Practical chemotherapy demonstration with appropriate Personal Protective Equipment and celebrating the first chemotherapy administration with appropriate precautions in a regional hospital post-training. Source: Left panel by Jayne Harrison, Children’s Cancer Centre, Royal Children’s Hospital, Melbourne, Victoria, Australia; Murdoch Children’s Research Institute, Global Health Research, Melbourne, Victoria, Australia. Right panel by Ann Williams, East New Britain Provincial Hospital, Papua New Guinea. Used with permission from both authors and the individuals in the photo.

Key activities were planned in line with the above visit goals and undertaken by the in-country health care team in partnership with the visiting delegation (**Table 1**). The delegation toured PMGH, assessing staff education needs, their facilities and access to resources. Practical demonstrations included familiarisation with and interpretation of oncology treatment protocols, safe chemotherapy handling and administration (**Figure 1**), comprehensive bedside nursing assessment of patients, and inclusive and sensitive delivery of family education and advice. The workshop attracted 41 participants and achieved national reach (attendees travelled from five additional Provincial Health authorities outside of Port Moresby, with support from the PNG National Department of Health Cancer Unit).

**Table 1:** Key activities, achievements and learnings

Activity	Details	Achievements	Learnings
Goal 1: provide education and training to upskill the workforce caring for children with cancer in PNG			
*Two-day multidisciplinary workshop*	Topics addressed: 1) Global Initiative for Childhood Cancer – update on progress, 2) introduction to leukaemia care and adapted treatment regimens, 3) focus on retinoblastoma as a WHO Index cancer – awareness and early detection, 4) early diagnosis of solid tumours; 5) chemotherapy – dosing, safe handling, administration and complications, 6) supportive care, 7) palliative care.	High attendance at the workshop (n = 41), including nurses, medical doctors, pharmacists, social workers and chaplains. Attendees included representation from five additional Provincial Health Authorities. A highly positive evaluation from attendees. Learning activities were aligned with learning outcomes and local experience, considering principles of adult learning theory, including experiential learning and social constructivism. Given the varied knowledge and skill set of attendees, including experienced chemotherapy providers and chemotherapy-naive staff in small centres, the workshop was able to promote scaffolded peer-to-peer learning and build rapport between staff from different professions and centres.	Infrastructure, particularly technology (*e.g.* stable internet), is critical to building and maintaining an effective childhood cancer care program. The use of breakout groups and simulation for practical skills training enabled a high level of participant engagement and opportunities for practice aimed to enhance learning retention. A focus on interprofessional education generated an environment of mutual respect and open communication, leading to a focus on interdisciplinary collaborative practice, which has the potential to strengthen local care capacity.
Goal 2: develop and implement a PNG Childhood Cancer Database to enable effective patient management and accurate data collection and analysis			
*Launch the PNG Childhood Cancer Database*	Officially launched the PNG Childhood Cancer Database. Provided training to clinical and administrative staff on accurate data extraction and entry.	Successfully commenced data extraction and data entry. Tailored the Redcap database as appropriate to address unique aspects of patient data in PNG. Examples include adding additional ethnicity options and languages (PNG has >800 recognised languages), free textboxes to describe village landmarks for families with no street address, and including place of birth for parents because many people in PNG use a single name.	Administrative staff who are well-trained and protected from clinical duties are critical for successful data entry and ongoing database maintenance.
Goal 3: deliver ward rounds and provide clinical education to improve care for current patients			
*Clinical support on the ward*	Multidisciplinary ward rounds were performed in combination with in-country staff and delegates, and there were opportunities for close discussion with family members.	Provision of clinical expertise in diagnostic reasoning and treatment decisions including chemotherapy and palliative care. Active teaching and practical demonstrations illustrated potential learning opportunities and the educational value of ward rounds. Delegates obtained a true and meaningful insight into the clinical environment and thus could appropriately target further teaching and support. A strong medical and nursing leadership provided excellent clinical care, compounded by limitations including rotating junior staffing, limited space, and medication availability.	Due to prolonged hospital admissions and staffing constraints, parents of paediatric oncology patients in PNG play a large role in their clinical care. Further education and resources are needed for this important group. A large proportion of current inpatients and families are clinically stable and safe for outpatient therapy, however, with no access to local accommodation for their vulnerable and immunocompromised children.
*Collaboration with interdisciplinary staff*	Roundtable with hospital-based chaplains to discuss pastoral care needs of children with cancer. Meeting with a hospital-based social work team. Meeting and tour of pharmacy department.	There is a high level of engagement from hospital chaplains, with attendance from multiple chaplains. Chaplains expressed eagerness to continue professional development in providing care for families, regardless of their faith affiliation. Enthusiastic and engaged response from the social work team, who expressed their challenges and passion for working with families of childhood cancer patients. Direct review of current chemotherapy safety practices and needs, as well as further insights into issues with current chemotherapy supply.	Hospital-based chaplains play a vital role in the health system in PNG, as confirmed in the IMPACT review. Future education programs should engage chaplain service early and authentically. Social workers in PMGH cover a multitude of roles, including providing urgent social support and welfare for women in crisis, thus having limited capacity for childhood cancer families. There is a strong, enthusiastic hospital pharmacy team with a passion for improving local procedures with support.
Goal 4: engage key stakeholders to advocate for more resources for children with cancer in PNG			
*Engagement with hospital leadership, Ministry and WHO in-country office*	Meetings with stakeholders included nursing leaders from the PMGH and regional provincial hospitals, the Director of Nursing, the Health Ministry and WHO representatives.	Critical to the success and sustainability of the program, we were able to demonstrate robust support and collaboration with local medical and nursing leadership and the SIOP Oceania team, setting up well for further advocacy work. These meetings were key opportunities to highlight the importance of a trained and educated nursing and medical workforce to support the care of children diagnosed with cancer in the region.	The leadership recognised the importance and need for further investment into this area of health care in their region. The Director of Nursing expressed the need for assistance with the development of a specific oncology nursing program.
Goal 5: provide practical resources to improve care for children with cancer in PNG			
*Resources provided during the visit*	Books/toys, spill kits, retinoblastoma awareness posters, prosthetic eye toy elephant, comfort first equipment, USBs with presentations, eye patches, toothbrushes, thermometers, parent information resources	The resources provided complemented the education sessions and were targeted at the participants’ highlighted areas of need, namely early diagnosis within the community, parent education, and palliative care. A ‘train the trainer’ approach was used for parent education in particular, with nursing staff expressing a keen desire to continue this with the resources provided.	Nursing staff, both from regional and primary oncology workplaces, were passionate about improving patient care, including placing value on parent education. Resources are required to be adapted to the local setting, including the use of photos and stories as guided by the local team.
*Resources provided after the visit*	Following the visit, additional resources were sent to PNG including digital thermometers, toothbrushes, eye patches, ‘Ollie the Elephant’ with a prosthetic eye, and books (*e.g.* The Invisible String, patient diaries). The sharing of these resources continues.	Ongoing resource sharing has been one component of ongoing collaboration and strengthening of relationships between the two teams. Examples include the development of adapted resources, such as a family education booklet entitled ‘Caring for your child at home following a cancer diagnosis in PNG’ and a nursing guide entitled ‘Nursing care for patients receiving chemotherapy in PNG.’	A longitudinal approach is required for effective capacity-building partnerships. Nurturing of established professional relationships.

IMPACT – Integrated Mission of Programme of Action of Cancer Therapy, PMGH – Port Moresby General Hospital, PNG – Papua New Guinea, SIOP – International Society of Paediatric Oncology, USB – universal serial bus, WHO – World Health Organization

## SUSTAINING A COMMITMENT TO BUILDING NURSING CAPACITY

Specialist oncology nursing is the cornerstone of modern child cancer care, but developing and sustaining a workforce with specific training in cancer care is a major challenge in countries such as Papua New Guinea, where the majority of hospital-based nursing is generalist, and few opportunities exist for the development of advanced care or specialist practice. Through the relationships forged during the visit, Papua New Guinea nurse participants expressed a strong desire and keen interest in progressing their skills, competencies and nursing practice.

This enthusiasm and support led to the development of two strategies. First, the establishment of a dedicated national oncology ‘community of practice,’ the Papua New Guinea Oncology Nurses Association (PaNGONA) via a WhatsApp group, to sustain support from facilitators, foster connections, and provide peer support and share experiences between nurses. PaNGONA is now a registered organisation with the Papua New Guinea Investment Promotion Authority with a formal constitution and a strategic plan. It includes a SIOP Oceania nursing delegate to the advisory board. Membership of PaNGONA has rapidly grown to 183 nurses caring for adults and children with cancer nationally. Second, paediatric oncology nursing education meetings and teaching sessions are held monthly via Zoom, covering requested topics, including chemotherapy administration, supportive care, and cancer-specific education.

## OBSERVATIONS AND RECOMMENDATIONS FOR CAPACITY BUILDING IN SMALL ISLAND NATIONS SUCH AS PAPUA NEW GUINEA

During and after the delegation, the in-country team identified a need for increased focus on palliative care, which is particularly critical in lower-resourced settings. In Papua New Guinea, palliative care delivery challenges can be further compounded by unique spiritual beliefs, which can impact local acceptance of medical diagnoses and prognostication [9]. In response, supported by philanthropy, SIOP Oceania will co-develop and implement palliative care education and services in the region in 2024–25.

Additional capacity-building components include establishing a formal multidisciplinary team meeting, including pathology and surgical staff, developing an adapted treatment regimen, further education, including a follow-up in-country visit/workshop, fundraising for further nursing and medical clinical observerships in Australia, and ongoing advocacy in close partnership with local clinicians.

Partnerships between medical institutions in high-income countries and LMICs are known to improve paediatric cancer control efforts, with multidisciplinary team visits to LMICs being one reported key factor in partnership success [10]. Communities of practice are effective tools for teaching and knowledge dissemination, thus contributing to capacity building to improve local patient care [11]. The partnership evolution between the PMGH Paediatric Oncology team and SIOP Oceania and the subsequent formation of local collaborations (particularly PaNGONA) has demonstrated the impact of supportive peer relationships in global oncology. This is especially vital in the geographical setting of Papua New Guinea, with multiple care providers working in relative isolation in small provincial centres. Our partnership has thus enabled further opportunities for education and standardisation of diagnostic and therapeutic approaches nationwide. However, challenges in Pacific Island countries extend beyond health care infrastructure and include social, environmental and public health factors that require multi-faceted interventions to address population needs.

Although there was a long pre-existing relationship between SIOP Oceania and the local team, there were significant new lessons learned during the in-country visit. The visit highlighted key features of Papua New Guinea culture, including a strong value for respect/hierarchy, which initially posed challenges to the planned interactive workshop delivery. These challenges were overcome with the development of social peer relationships and the fostering of a psychologically safe environment in smaller group settings. The strong Christian faith in the community and, thus, the important role of chaplains in hospital care was also a key learning. The high level of chaplain participation in education sessions created further diversity of participants and provided a distinct perspective on opportunities to enhance care, especially for patients requiring palliative care.

Finally, the role of local champions is paramount in creating successful partnerships and ongoing capacity building. Ultimately, we have found that it ‘takes a multi-disciplinary village’ to strengthen cancer care delivery for children in Papua New Guinea, particularly with our partnership between in-country and visiting health professionals comprising a complementary multidisciplinary skillset.

## References

[1] Gupta S, Howard SC, Hunger SP, Antillon FG, Metzger ML, Israels T, et al. Treating Childhood Cancer in Low- and Middle-Income Countries. In: Gelband H, Jha P, Sankaranarayanan R, Horton S, editors. Cancer: Disease Control Priorities, Third Edition (Volume 3). Washington D.C., USA: The International Bank for Reconstruction and Development/The World Bank; 2015. p. 121–46. 26913338

[2] Sarfati D Dyer R Sam FA-L Barton M Bray F Buadromo E Cancer control in the Pacific: Big challenges facing small island states. Lancet Oncol. 2019;20:e475–92. 10.1016/S1470-2045(19)30400-0 31395476 PMC7746436

[3] World Health Organization. UHC Service Coverage Index (S.D.G. 3.8.1). Geneva, Switzerland: World Health Organization; 2019. Available: https://www.who.int/data/gho/data/indicators/indicator-details/GHO/uhc-index-of-service-coverage. Accessed: 21 February 2024.

[4] World Health Organization. Global Health Observatory Database: Global Health Workforce Statistics. 2023. Available: https://apps.who.int/gho/data/node.main.HWFGRP?lang=en. Accessed: 21 February 2024.

[5] Ward ZJ Yeh JM Bhakta N Frazier AL Atun R Estimating the total incidence of global childhood cancer: a simulation-based analysis. Lancet Oncol. 2019;20:483–93. 10.1016/S1470-2045(18)30909-4 30824204

[6] Das M New imPACT review in Papua New Guinea. Lancet Oncol. 2023;24:1306. 10.1016/S1470-2045(23)00562-4 39491840

[7] Daur B. Outcomes of paediatric cancers in Port Moresby General Hospital. 2018. Available: https://pngpaediatricsociety.org/wp-content/uploads/2018/09/Benamin-Daur-Outcomes-of-paediatric-cancer-in-PNG-DCH-2018.pdf. Accessed: 22 February 2024.

[8] Kiromat M Vince J Oswyn G Tefuarani N The management of children with cancer in Papua New Guinea: a review of children with cancer at Port Moresby General Hospital. P N G Med J. 2004;47:138–45. 16862938

[9] Siddiqui MF Nohra L Saleh M Thakkar K Trivedi R Moujally SN Pediatric Oncology, Palliative Care and Low- or Middle- Income Countries: A Call for Action. Glob Pediatr Health. 2023;10:X2333794X231188591. 10.1177/2333794X231188591 37492651 PMC10363861

[10] Ribeiro RC Antillon F Pedrosa F Pui CH Global pediatric oncology: lessons from partnerships between high-income countries and low-to mid-income countries. J Clin Oncol. 2016;34:53. 10.1200/JCO.2015.61.9148 26578620 PMC4980571

[11] Ranmuthugala G Plumb JJ Cunningham FC Georgiou A Westbrook JI Braithwaite J How and why are communities of practice established in the healthcare sector? A systematic review of the literature. BMC Health Serv Res. 2011;11:273. 10.1186/1472-6963-11-273 21999305 PMC3219728

